# The magnitude of the germinal center B cell and T follicular helper cell response predicts long-lasting antibody titers to plague vaccination

**DOI:** 10.3389/fimmu.2022.1017385

**Published:** 2022-10-28

**Authors:** Darrell R. Galloway, Nguyen X. Nguyen, Jiahui Li, Nicholas Houston, Gage Gregersen, E. Diane Williamson, Frank W. Falkenberg, James N. Herron, J. Scott Hale

**Affiliations:** ^1^ Department of Molecular Pharmaceutics, University of Utah, Salt Lake City, UT, United States; ^2^ Department of Pathology, Division of Microbiology and Immunology, University of Utah, Salt Lake City, UT, United States; ^3^ Chemical Biological Radiological Division, Defense Science and Technology Laboratory (DSTL) Porton Down, Salisbury, United Kingdom; ^4^ CyTuVax B.V., Maastricht, Netherlands

**Keywords:** T follicular helper cell, germinal center, B cell response, antibodies, Yersinia pestis (plague), vaccination, subunit vaccine, immunological memory and vaccines

## Abstract

The development of a safe and effective vaccine against *Yersinia pestis*, the causative organism for plague disease, remains an important global health priority. Studies have demonstrated effective immune-based protection against plague challenge that is induced by plague antigen subunit vaccination in an aqueous alhydrogel formulation; however, whether these candidate vaccines in this formulation and presentation, induce long-lasting immunological memory in the form of durable cellular and antibody recall responses has not been fully demonstrated. In this study, we analyzed germinal center T follicular helper and germinal center B cell responses following F1V and F1 + V plague subunit immunization of mice with vaccines formulated in various adjuvants. Our data demonstrate that recombinant plague protein immunization formulated with IL-2/GM-CSF cytokines bound to alhydrogel adjuvant drive an increase in the magnitude of the germinal center T follicular helper and germinal center B cell responses following primary immunization, compared to vaccines formulated with Alhydrogel adjuvant alone. In contrast, plague protein subunit immunization combined with CpG ODN bound to alhydrogel increased the magnitude and duration of the germinal center Tfh and B cell responses following booster immunization. Importantly, enhanced germinal center Tfh and B cell responses correlated with long-lasting and high F1V-specific antibody titers and more robust antibody recall responses to F1V re-exposure. These findings indicate that vaccine formulations that drive enhancement of the germinal center Tfh and B cell responses are critical for inducing durable plague-specific humoral immunity.

## Introduction

The generation of specific, effective and long-lasting antibody responses are a central goal of vaccination to induce lasting immunological memory that can prevent disease caused by infectious microorganisms. For decades, efforts to generate safe and effective vaccines that provide protection against plague have been pursued. Several plague subunit vaccines formulated in alhydrogel alone as an adjuvant have undergone clinical trials, but these have not yet progressed to full approval (1, 2). One major barrier to progress in this area is that the generation of long-lasting immunity and robust antibody recall responses have not been formally demonstrated. Given that plague subunit vaccination when adjuvanted by alhydrogel alone is relatively weak (2–5), it is unclear whether such vaccine formulations with alhydrogel alone are sufficient to induce long-lasting memory and robust recall responses upon rechallenge with antigen. Finding ways to enhance the durability of plague vaccine-induced antibody responses, is critical to the development of an effective vaccine and will contribute to improved knowledge for other vaccine programs as well.

Plague is an ancient disease that, along with smallpox, malaria, and tuberculosis, is among the top deadly infectious diseases throughout human history (6–10). Indeed, the three largest plague pandemics alone (Justinian, the Black Death and the Indo-China plague) account for greater than 200 million human deaths (11). The causative organism, *Yersinia pestis*, is a Gram-negative bacillus which is endemic in many parts of the world and periodically causes outbreaks resulting in the rapid accumulation of deaths before effective detection and treatment can be put in place. Plague remains a threat to vulnerable populations, recently demonstrated by an outbreak in Madagascar in 2017 in which 2,348 cases were reported and resulted in 202 deaths before being brought under control (12). In addition to the natural threat posed by this endemic pathogen, there has been a well-documented history of the use of *Y. pestis* as a biological weapon, an potential threat which remains in place today (8, 13, 14).

*Y. pestis* infects numerous animal hosts, while the common flea (*Xenopsylla cheopis*) serves as an intermediate host (15). The disease is transmitted to humans *via* the bite of an infected flea, and the ensuing disease manifests itself in two forms – bubonic plague which may proceed to a septicemic state, or a pneumonic form which is rapidly fatal if not diagnosed at a very early stage, and which becomes transmissible between humans and can lead to pandemics (16). The WHO has recently called for the development of an effective plague vaccine to address the urgent need for a protective vaccine against both bubonic and pneumonic forms of plague (17). There is currently no licensed vaccine available for plague in spite of numerous attempts at development (18–20).

Several animal model studies (21–23) have demonstrated that two virulence factors of *Y. pestis* appear to be promising target antigens for a protective immune response: the F1 capsular antigen (24) and the low calcium response LcrV (or V) antigen (19, 25–27). Two subunit vaccines based upon immunization with either the separate rF1 and rLcrV antigens (2, 8, 23) or a recombinant rF1V fusion protein (28) formulated in Alhydrogel generate humoral immune responses that are protective against both bubonic and aerosol plague challenges in animal models and have been evaluated in clinical trials (2). There have been several studies conducted focusing on vaccine strategies and formulations, including the use of various adjuvants, to improve the immune response against the F1 and LcrV antigens (10, 29). While the results of these studies have shown that various adjuvants are capable of enhancing the immune response (antibody), none have provided insight into the basis for the adjuvant effects nor a strategy to improve upon the induction of long-term immunological memory. Given the difficulty of plague vaccine candidates to generate long-lasting humoral responses, further studies are needed to provide additional insights into the T cell and B cell subsets that contribute to long-lasting vaccine induced immune protection.

The production of effective, long-lasting antibody responses requires the formation of the germinal center (GC), anatomical structures within lymphoid organs such as lymph nodes, where antigen-activated B cells undergo somatic hypermutation and selection that drives antibody affinity maturation and long-lived memory B cell and plasma cell differentiation (30). GC B cells require help from T follicular helper cells (Tfh), a subset of CD4+ T cells which are identified by their expression of the follicle homing chemokine receptor CXCR5 and Bcl6, a transcription factor that is required for Tfh cell differentiation (31–33). Tfh cells provide help to GC B cells *via* their delivery of IL-21, IL-4 and CD40L signals to facilitate GC B cell selection and memory B cell and long-lived plasma cell differentiation (30, 34). A subset of Tfh cells with high Bcl6 expression and that are localized within the germinal center, termed GC Tfh cells, express the highest levels of Il-4 and IL-21 (35). Importantly, increasing magnitude and/or quality of GC Tfh cells and GC B cell responses drives improved antibody responses following immunization (36–39).

While extensive studies have sought to identify ways to induce effective cellular and humoral responses for plague vaccination, analysis of Tfh and B cell responses within the germinal center have not been investigated. Given the importance of the GC T and B cell response for effective immunization and induction of protective and long-lasting antibody responses, combined with the relatively short-lived responses induced by plague vaccine candidates, we sought to evaluate the quality, magnitude, and duration of the GC Tfh and B cell responses following plague subunit vaccination in combination with various adjuvants. In addition to the standard formulation of alhydrogel as an adjuvant, we selected to test CpG ODN 1826 based on recent studies demonstrating its efficacy in vaccine-induced protection against plague challenge (29). We also evaluated the effects of cytokine-based formulation in combination with alhydrogel that included GM-CSF and IL-2 (40). We hypothesized that increased Tfh and GC B cell response will lead to improved long-lasting anti-plague antibody responses. In this study, we immunized mice with recombinant plague antigens formulated with several types of adjuvants including Alhydrogel, Alhydrogel combined with CpG ODN 1826, and Alhydrogel combined with IL-2 + GM-CSF. We evaluated the Tfh cell and GC B cell responses in the draining lymph nodes following primary immunization and boosting and evaluated the long-lived antibody responses. Our findings demonstrate that IL-2/GM-CSF cytokine-based nanoparticle adjuvants enhanced the initial magnitude of the GC response, while CpG-formulated plague vaccines induce more robust and longer-lasting Tfh and GC responses following booster immunization. These altered GC responses in IL-2/GM-CSF cytokine and CpG formulated vaccines correlated with improved long-lasting vaccine-induced F1V specific antibody responses, and significantly enhanced antibody recall responses upon re-exposure to antigen when compared to the conventional alhydrogel-formulated vaccines. We propose that vaccine formulations that combine either IL-2/GM-CSF cytokines or CpG with alhydrogel generate more durable GC center Tfh and B cell responses following plague subunit vaccination that lead to enhanced durability to the protective antibody response.

## Material and methods

### Plague vaccine formulations and animal immunization

The purified recombinant plague vaccine antigen, rF1V, was kindly provided through the Joint Program Executive Office (JPEO-CBRND, Aberdeen, MD) through the U.S. Department of Defense and has previously been described (41). The rF1 (F1), and rLcrV (V) antigens were expressed from *E. coli* and purified as previously described (2). Antigens were formulated with three separate adjuvants in order to evaluate whether there is any immunological enhancement following immunization. The adjuvants formulations included: 1) Alhydrogel (*In vivo*Gen); 2) a cytokine mixture (rhIL-2 and rmGM-CSF – Peprotech) also combined with Alhydrogel; and 3) CpG 1826 (*In vivo*Gen) also combined with Alhydrogel. Vaccine composition per dose was as follows: 1) Alhydrogel group: 10 μg antigen [either rF1V or 10 μg each rF1 and rV] + 30 μg Alhydrogel per 0.1mL dose; 2) cytokine group:10 μg antigen + 30 μg Alhydrogel + 10 μg IL-2 and 10 μg GM-CSF per 0.1 ml dose; 3) CpG group: 10 μg antigen + 30 μg Alhydrogel + 50 μg CpG 1826 per 0.1 ml dose. Female Balb/c mice were purchased from Jackson Laboratories, and at 10 weeks of age were immunized intramuscularly (i.m) with a 50 μl injection into the quadricep of each hind leg for a total volume of 100 μl per mouse. They were immunized on day 0 and given booster shots on day 16. On day 126, the remaining mice were each challenged with 100 μl of F1V fusion protein (10 μg rF1V in PBS and no adjuvant) *via* intraperitoneal (i.p) injection. Each vaccine group for each individual timepoint within an experiment consisted of 5 mice per group and experiments were performed at least two times. For analysis of germinal centers, the draining lymph nodes (pooled 2 inguinal plus 2 lumbar) were harvested on days 7, 14, 23, and 30. All animal experiments were conducted in accordance with approved University of Utah IACUC protocols.

### Tissue preparation/blood collection

Inguinal and lumbar (draining) lymph nodes and spleens were dissected on days when mice were sacrificed. Single-cell suspensions of draining lymph nodes and spleen were prepared by smashing the tissues through a 70 μm cell strainer. Cells collected by centrifugation and resuspended in RPMI media + 5% FBS. Red blood cells were lysed from spleen cell suspensions using the Ack lysis buffer (Gibco). Blood was collected *via* the submandibular vein.

### ELISA immunoassay

F1V, F1, and V specific serum IgG, IgG_1,_ and IgG_2a_ titers were determined by ELISA as previously described (22). In brief, recombinant proteins were coated onto microtiter plates (Immulon 2HB, Fisher) at a final concentration of 5 μg/ml by overnight incubation at 2-8 °C, followed by washing in PBS, 0.02% Tween 20 and blocking with PBS and 1% skim milk for one hour. Serum samples were tested in triplicate in a series of two-fold dilutions. Each plate incorporated a series of standards for determining the final antibody concentration curve for each plate, which included a standard antibody capture assay in which goat anti-mouse IgG (Fab) (Sigma) was used to capture purified mouse IgG standards (Sigma) in a series of two-fold serial dilutions such that a standard mouse IgG binding curve could be determined for each plate (22). Following the washing step, each plate was developed by addition of a secondary goat anti-mouse IgG (_H&L_) labeled with horse radish peroxidase (HRP) for one hour at 37 °C, followed by the addition of a fluorogenic peroxide substrate (QuantaBlue). The reaction was stopped after 20 min, and the plates were scanned at 420 nm using Biotek Synergy H1M plate reader. The data was recorded using the BioTek Gen5 software program and analyzed using an Excel spreadsheet program. Titers were calculated as μg anti-F1V antibody per ml of serum for comparative analysis. Serum samples for analysis were collected on days 0, 14, 23, 30, 126 and 133.

### FACS analysis and activation induced marker (AIM) assay

For flow cytometry, 1–3 × 10^6^ cells from single-cell suspensions of draining lymph nodes or spleen were transferred to 96-wells round-bottom plates for subsequent antibody staining. Surface staining for flow cytometry used fluorochrome-conjugated antibodies: B220, CD138, IgD, CD4, PD-1, CD44 (Biolegend); CD19; Fas (BD), or with FITC- or Biotin-labelled peanut agglutinin (PNA). CXCR5 staining was performed as described previously (42) using purified rat anti-mouse CXCR5 (BD) for 40 minutes, followed by a secondary Biotin-SP-conjugated Affinipure F(Ab’)2 goat anti-rat IgG (Jackson Immunoresearch) for 30 minutes, and then followed with APC- or PeCy7-labeled streptavidin (eBioscience) (31). For transcription factor staining, cells were surface stained, followed by permeabilization and fixation, and then stained for Bcl6 (BD) and Tbet (Biolegend) using the Foxp3/Transcription Factor Staining Buffer Set and protocol (eBioscience). Flow cytometry data were collected on a FACS Canto II, FACS Fortessa and X20 (BD). FACS data were analyzed using FlowJo 10 software (TreeStar Inc.) For the Activation-induced marker (AIM) assay, 1x10^6^ cells were transferred to 96-well flat-bottom plates for stimulation. Two hundred μl of RF10 media (RPMI supplemented with FBS 5%, 1% L-glut, 1% Pen/Strep, 50mM BME) either with or without protein antigen were added to each well. For antigen re-stimulation, 5μg of F1V antigen in PBS was added per 1 mL of RF10 media. Cells were then incubated for 18 hours at 37°C and 5% CO_2_ before getting transferred to round-bottom plates for appropriate antibody staining (43, 44). To identify Ag-specific CD4+ T cells, cells were stained with the following panel: CD4, B220, PD-1, CD25, OX40, PD-L1 (Biolegend); CXCR5, Foxp3 (eBio); and Bcl6, CD154 (BD).

### Statistical analysis

Statistical analyses were performed using Graphpad Prism 9. Statistically significant p values of <0.05 are indicated and were determined using a two-tailed unpaired Student’s t test and Two-way ANOVA.

## Results

### CpG and cytokine adjuvants induce robust GC Tfh cell responses to plague antigen vaccination during the first seven days of the primary immune response

**Figure 1:**
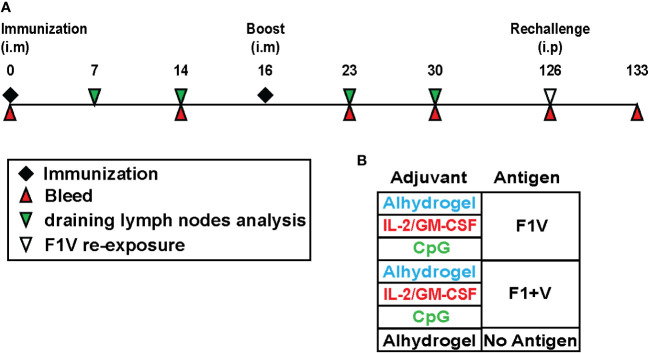
Recombinant plague antigen vaccine study design to evaluate germinal center B cell and T cell responses and long-lived antibody responses. **(A)** Schematic showing the study timeline and timepoints for immunization, draining lymph node collection, and blood collection from immunized mice. Mice were immunized and boosted intramuscularly with antigen and adjuvant at day 0 and day 16. At day 126, mice were injected intraperitoneally with F1V antigen (no adjuvant) and the antibody recall response was measured 7 days later (day 133). **(B)** Table shows the various antigen + adjuvant vaccine groups.

To evaluate adjuvant and recombinant plague antigen combinations for their capacity to induce Tfh cell and germinal center B cell responses in mice, we immunized mice intramuscularly (i.m.) and boosted 16 days later with the corresponding vaccine preparation. The adjuvant combinations tested here were Alhydrogel, Alhydrogel plus a combination of IL-2 + GM-CSF (referred to hereafter as cytokines), and Alhydrogel plus CpG 1826. Mice were sacrificed 7, 14, 23, 30 post-immunization to collect draining lymph nodes (dLNs - a pool of 2 inguinal and 2 lumbar lymph nodes) to evaluate T cell and B cell responses (**Figures 1A, B**).

**Figure 2:**
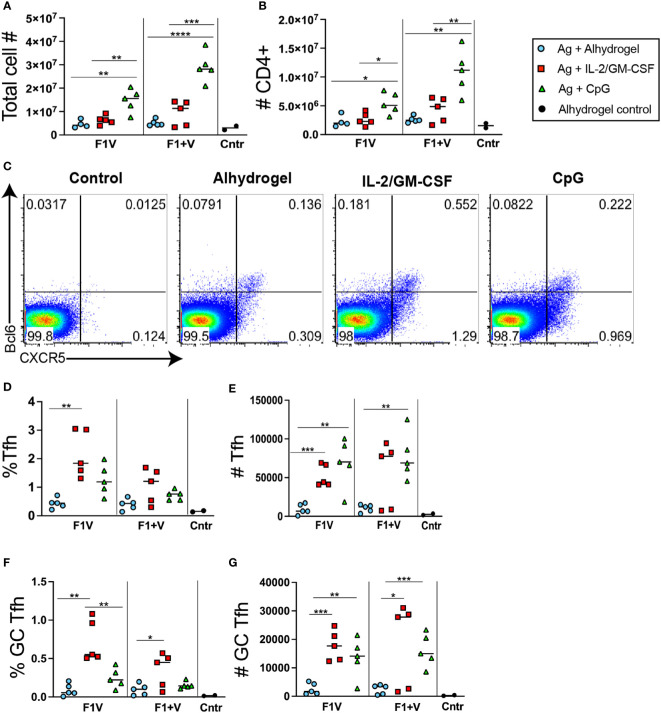
T follicular helper cell responses 7 days post-immunization. Analysis of lymphocyte responses in the draining lymph nodes (dLNsj) 7 days post-immunization. **(A)** Total number of cells in dLNs. **(B)** Total number of CD4+ T cells in dLNs. **(C)** Flow cytometry analysis of CXCR5 and Bcl6 expression by CD4+ gated T cells in the dLNs. **(D)** Frequency and **(E)** number of Tfh (CXCR5+) cells in the dLNs. **(F)** Frequency and **(G)** number of CXCR5+ Bcl6+ GC Tfh in dLNs. n=5 per group per experiment at each time point. Data shown are from one experiment and are representative of two independent experiments. Statistically significant p values were determined using a two-tailed unpaired Student t test; *p < 0.05, **p < 0.01, ***p < 0.001, ****p < 0.0001.

Analysis of draining lymph nodes seven days post-immunization revealed that mice immunized with CpG 1826 formulated vaccines had significantly higher numbers of total lymphocytes and CD4+ T cells in the dLNs (**Figures 2A, B**). Flow cytometry analysis of CD4+ T cells revealed the populations of Tfh cells (CXCR5+ in upper right + lower right quadrants) and GC Tfh cells (CXCR5+Bcl6+ in upper right quadrant), and these subsets were elevated compared to control mice (adjuvant only but no antigen) (**Figure 2C**). Although the percent of CD4+ T cells that are Tfh and GC Tfh cells in the CpG 1826 adjuvanted group is similar (or trending higher) to that seen in the antigen + alhydrogel formulated vaccine groups (**Figures 2D, F**), when factoring in total cell numbers in the dLNs (**Figure 2A**) and number of CD4+ T cells (**Figure 2B**), both CpG 1826 immunized and IL-2/GM-CSF cytokine formulated vaccine groups of mice exhibited significantly higher numbers of Tfh cells and GC Tfh cells than alhydrogel-only formulated vaccine groups (**Figures 2E, G**). Interestingly, GC Tfh cells from CpG-formulated vaccine groups expressed significantly higher levels of the Th1-associated transcription factor Tbet, suggesting that CpG induces a modest Th1-like skewing of Tfh cells induced by vaccination (**Supplementary Figure 1**). Together, these data show that both F1V and F1 plus V antigens when formulated with Alhydrogel-bound IL2+GM-CSFcytokines or CpG 1826 generate significantly increased GC Tfh cell responses 7 days post-immunization compared to protein vaccination with the alum-based Ahydrogel adjuvant alone.

**Figure 3:**
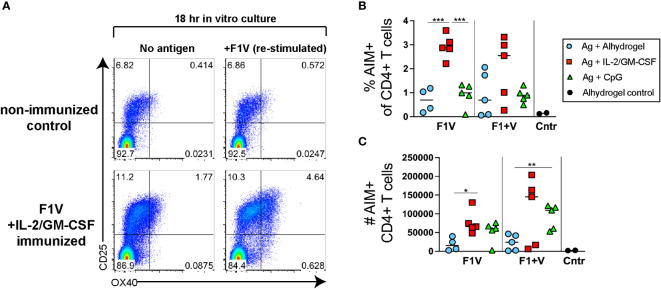
Plague antigen F1V-specific CD4+ T cells as detected by activation-induced marker (AIM) assay. Cells from the draining lymph nodes 7 days post-immunization from immunized mice were cultured for 18 hours in the presence or absence of recombinant F1V antigen. Cells were then stained for surface markers and evaluated for antigen-induced upregulation of both OX40 and CD25. The frequency of specific AIM+ cells (% AIM+ of CD4+ T cells shown in **Figure 2B**) was calculated as the frequency of CD4+B220- T cells that specifically express both OX40 and CD25 (OX40+CD25+ double-positive population in upper right quadrant) cells following 18 hours of culture in the presence of F1V antigen minus the background frequency of OX40+CD25+ cells from the same sample following 18 hour culture in the absence of antigen. **(A)** Representative flow cytometry analysis of OX40 and CD25 expression by CD4+ gated T cells, of a control mouse (injected with alhydrogel but not immunized with antigen) and a F1V + IL-2/GM-CSF cytokine immunized mouse. **(B)** Chart shows the percent AIM+ of CD4+ T cells in the dLNs, which is the frequency of cells that are OX40+CD25+ cells in the F1V-restimulated minus the no-antigen culture for each individual sample. **(C)** Chart shows the number of AIM+ F1V-specific OX40+CD25+ CD4+ T cells in the dLN. n=5 per group per experiment at each time point. Statistically significant p values were determined using a two-tailed unpaired Student t test; *p < 0.05, **p < 0.01, ***p < 0.001.

To determine whether the Tfh cell responses observed (**Figure 2**) were comprised of antigen-specific CD4 T cells, we used an activation-induced marker (AIM) assay (43, 44). Lymphocytes from draining lymph nodes were re-stimulated for 18 hours *in vitro* with recombinant F1V antigen and compared to cells from the same sample that were cultured in the absence of antigen. The cultured samples were then assayed to determine the percent of CD4+ T cells that specifically upregulated both OX40 and CD25 (activation induced markers) following culture in the presence of F1V antigen compared to culture without antigen. In control mice injected with Alhydrogel alone but not immunized with antigen, the background percent of CD4+ T cells that are AIM+ cells (OX40hiCD25hi) following restimulation with F1V antigen in culture was relatively low (**Figures 3A, B**). In contrast, an average of ~3% of CD4+ T cells were AIM+ cells following re-stimulation in the F1V + IL-2/GM-CSF immunized mice (**Figures 3A, B**). Both IL-2/GM-CSF cytokine and CpG 1826 formulated vaccine groups of mice produced a robust number of F1V-specific CD4+ T cells, while the Alhydrogel groups had significantly lower numbers of F1-V-specific CD4+ T cells (**Figures 3B, C**). These data demonstrate that the AIM assay can be used to quantify F1V-specific CD4 T cell responses in draining lymph nodes following plague subunit protein vaccination.

**Figure 4:**
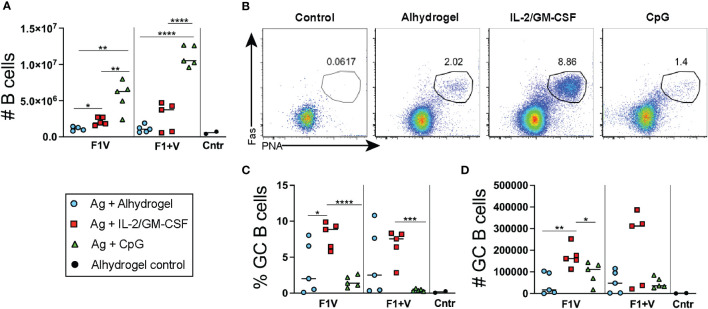
Germinal center B cell responses 7 days post-immunization. Analysis of lymphocyte responses in the draining lymph nodes (dLNs) 7 days post-immunization. **(A)** Total number of CD19+ cells in dLNs. **(B)** Flow cytometry analysis of CD19+ gated B cells with gate showing PNA+Fas+ GC B cells. **(C)** Frequency and **(D)** number of GC B cells. n=5 per group per experiment at each time point. Data shown are from one experiment and are representative of two independent experiments. Statistically significant p values were determined using a two-tailed unpaired Student t test; *p < 0.05, **p < 0.01, ***p < 0.001, ****p < 0.0001..

Seven days post-immunization, the CpG 1826 immunized groups of mice had significantly higher numbers of B cell in the dLNs compared to mice immunized with the IL-2/GM-CSF or alhydrogel-only vaccine formulations (**Figure 4A**). Analysis of B220+CD19+ gated B cells revealed a significantly higher frequency of PNA+Fas+ GC B cells present in the cytokine-formulated groups compared to Alhdrogel-only and CpG 1826 formulated groups (**Figures 4B–D**). Together, these data combine to demonstrate that Alhydrogel alone as an adjuvant for plague subunit vaccination generated relatively weak GC Tfh cell and B cell responses in the dLNs seven days post-immunization. In contrast, both IL-2/GM-CSF cytokine- and CpG-formulated vaccines enhanced GC Tfh cell numbers; however, only the cytokine-formulated vaccines induced significantly more GC B cells in the first week during the primary response.

### IL-2/GM-CSF cytokine adjuvants promote enhanced magnitude of the germinal center response after priming immunization, while CpG increases the duration of the germinal center response following booster immunization

**Figure 5:**
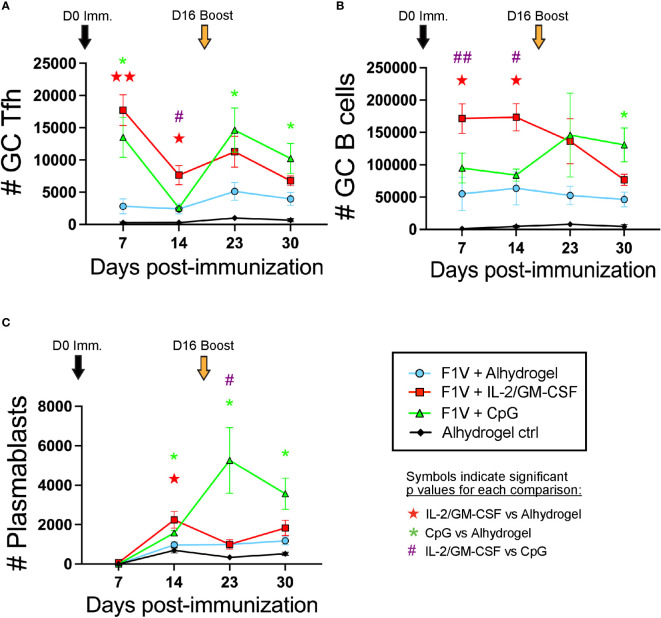
CpG and IL-2/GM-CSF as adjuvants differentially alter the magnitude and duration of the germinal center response following F1V primary and booster immunization. Mice were immunized on day 0 and given booster shots on day 16. Draining lymph nodes were collected for germinal center analysis on days 7, 14, 23, and 30 post-immunization. **(A)** Number of CXCR5+Bcl6+ GC Tfh cells. **(B)** Number of CD19+Fas+PNA+ GC B cells. **(C)** Number of CD19+IgD-CD138+ plasmablasts. n=5 per group per experiment at each time point. Data shown are from one experiment and are representative of two independent experiments. Statistically significant p values were determined using multiple unpaired Student t test; Colored symbols (red star, green asterisk, or purple hashtag) indicate significant p values for each of the indicated comparisons between experimental groups: one symbol for p < 0.05, two symbols for p < 0.01.

We analyzed the GC Tfh and GC B cell responses on days 7 and 14 following initial immunization, as well as 7 and 14 days following the booster immunization (days 23 and 30). The IL-2/GM-CSF cytokine-formulated group of mice exhibited significantly higher GC Tfh and GC B cell numbers at days 7 and 14 in the dLNs compared to the alhydrogel-only vaccine group (**Figures 5A, B**). In contrast, while the CpG 1826 formulated group also exhibited increased GC Tfh cells at day 7 compared to the Alhydrogel-only group, the GC Tfh cells in the CpG group of mice were decreased by day 14 and the GC B cell numbers were similar to those observed in the alhydrogel-only group at both days 7 and 14 (**Figures 5A, B**). Following booster immunization, both CpG 1826 and IL-2/GM-CSF cytokine formulated groups exhibited greater GC Tfh and GC B cell numbers compared to the alhydrogel-only vaccine group at day 23 (7 days post-boost) (**Figures 5A, B**). At day 30 (2 weeks post-booster), GC Tfh and GC B cell numbers in the IL-2/GM-CSF cytokine formulated group declined to numbers similar to those observed in the alhydrogel formulated vaccine group. In contrast, the CpG 1826 immunized mice maintained GC Tfh and GC B cell numbers at significantly elevated levels (compared to the alhydrogel group) through day 30 (**Figures 5A, B**). Interestingly, the plasmablast response in the dLNs was significantly elevated in both cytokine and CpG 1826-formulated groups compared to alhydrogel-only vaccine group at day 14 post-immunization; however, following boosting, plasmablasts in the CpG 1826-formulated group continue to rise and were significantly higher at days 23 and 30 (**Figure 5C** and **Supplementary Figure 2**). Together, these data show that mice immunized with plague subunit vaccines formulated with IL-2/GM-CSF have the highest early Tfh cell and GC B cell responses to primary immunization, while the CpG 1826 formulated groups induce elevated GC Tfh and GC B cell responses that persist significantly longer following the day 16 booster immunization.

### CpG ODN and IL-2/GM-CSF adjuvants drive enhanced and long-lasting antibody responses compared to vaccines formulated only with alhydrogel

**Figure 6:**
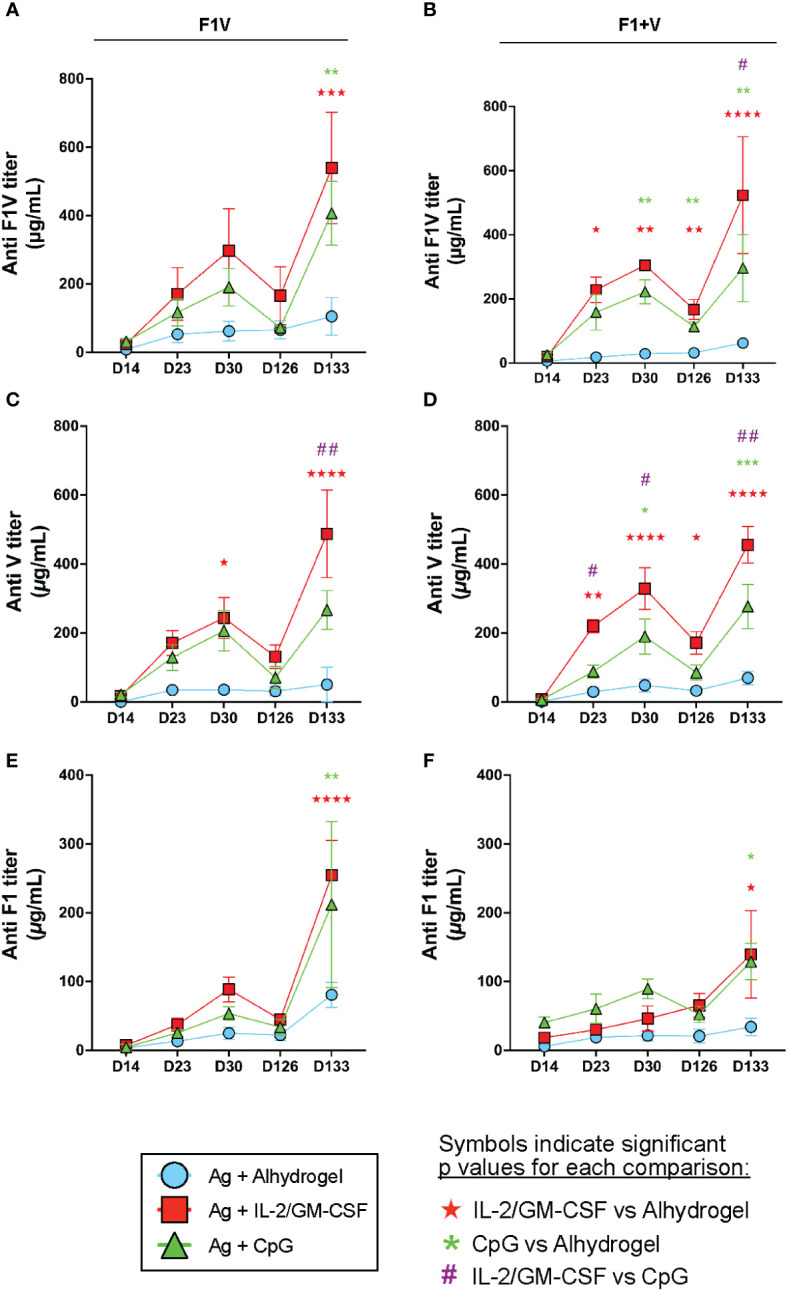
CpG and IL-2/GM-CSF as adjuvants drive increased long-lived antibodies against plague antigens. Mice were immunized on day 0 and given booster shots day 16. Remaining mice were challenged with 10 μg of F1V antigen on d126. Serum was collected on day 14, 23, 30, 126 and 133 for antibody titer analysis. Mean serum IgG titers of F1V antigen groups are shown for F1V **(A)**, V **(C)** and F1 **(E)**. Mean serum IgG titers of F1+V antigen groups are shown for F1V **(B)**, V **(D)**, F1 **(F)**. n=5 per group per experiment at each time point. Data shown are from one experiment and are representative of two independent experiments. Statistically significant p values were determined using Two-way ANOVA; Colored symbols (red star, green asterisk, or purple hashtag) indicate significant p values for each of the indicated comparisons between experimental groups: one symbol for p < 0.05, two symbols for p < 0.01, three symbols for p < 0.001, and four symbols for p < 0.0001.

To assess the magnitude and kinetics of the vaccine-induced antibody responses, we collected serum from immunized mice throughout the primary and boosted responses (**Figure 1A**). Priming and boosting with CpG- or IL-2/GM-CSF-formulated subunit vaccines resulted in increased F1V, V, and F1-specific antibody titers on days 23 and 30 (trending higher or in some cases significantly increased) compared to alhydrogel vaccine groups (**Figures 6A–F**). Importantly, at the day 126 memory timepoint, we observed that the F1+V immunized IL-2/GM-CSF cytokine group of mice had increased long-lasting F1V-specific and V-specific antibody titers compared to the Alhydrogel-formulated vaccine group (**Figures 6B, D**). In addition, at day 126, the F1+V CpG-formulated vaccine group exhibited significantly higher F1V-specific titers compared to the alhydrogel-only vaccine group (**Figure 6B**) and a trending increase in V-specific antibody titers (**Figure 6D**). Together, these findings indicate that compared to Alhydrogel only-formulated plague subunit vaccination, CpG 1826 and IL-2/GM-CSF cytokine-formulations drive improved long-lived antibody titers that persist at a memory timepoint.

To evaluate the antibody recall response to antigen re-exposure, we injected all groups of mice intraperitoneally at day 126 with 10 μg of F1V antigen (without adjuvant) and then measured antibody levels 7 days later (day 133 – see **Figure 1A**). We observed that mice immunized with Alhydrogel only-formulated F1V or F1 + V vaccination demonstrated negligible boosting of F1V, V, and F1-specific antibodies one week later (day 133) (**Figures 6A–F**). In contrast, mice immunized with plague antigens formulated with either Alhydrogel-bound CpG 1826 or IL-2/GM-CSF cytokines exhibited dramatic increases in their F1V, V, and F1-specific antibody titers that were higher than the Alhydrogel only-formulated vaccine groups (**Figures 6A–F**). In addition, the F1V-specific and V-specific antibody titers following recall response at day 133 were significantly higher in the IL-2/GM-CSF cytokine group compared to the CpG groups in F1+V immunized mice (**Figures 6B, D**), and significantly higher for anti-V specific titer in F1V-immunized mice (**Figure 6C**). Analysis of the immune response in the spleen at day 133 indicate that all experimental groups had similar GC Tfh cell responses 7 days post-re-exposure to F1V antigen, although there was a significantly smaller number of GC B cells in the mice that had been immunized previously with CpG-formulated vaccines (**Supplementary Figures 3A, B**). Together, these data clearly indicate that either the F1V fusion protein or the two component F1+V vaccines, when formulated with Alhydrogel-bound IL-2/GM-CSF cytokines, results in the highest plague antigen-specific antibody responses that are enhanced in both magnitude and longevity following prime and boost vaccination, and generate long-lasting immunity capable of robust antibody recall responses following antigen re-exposure. In addition, CpG-formulated vaccines generated dramatically improved antibody responses and capacity for robust antibody recall responses to antigen re-exposure compared to alhydrogel-alone formulated vaccines.

### IL-2/GM-CSF cytokine and CpG ODN adjuvants provide enhanced F1V-specific IgG2a in response to plague subunit vaccination

We examined the effect of the adjuvants upon the ratio of F1V-specific IgG1 and IgG2a titers at day 126. These results are summarized in **Table 1** and show that the relative abundance of F1V-specific IgG2a antibodies induced by either cytokine or CpG 1826 formulation is increased compared to alhydrogel-alone immunized mice (**Table 1**). In addition, both CpG and IL-2/GM-CSF groups of immunized mice exhibited skewing toward a higher IgG2a:IgG1 ratio of F1V-specific antibodies compared to mice immunized with antigen + alhydrogel only (**Table 1**).

**Table 1:** Increased IgG2a production in F1V + IL-2/GM-CSF cytokine and F1V + CpG immunized mice.

Vaccine group	Relative antibody titer		Ratio IgG2a/IgG1
	IgG1	IgG2a	
F1V + Alhydrogel	128	8	0.06
F1V + IL-2/GM-CSF	213	85	0.4
F1V + CpG	107	75	0.7

Antibody titers are reported as geometric means determined on day 126 post immunization. n=5 per group per experiment at each time point. Data shown are from one experiment and are representative of two independent experiments.

## Discussion

Despite the identification of plague antigens that can be effectively used to induce protective immunity against plague infection, the development of plague subunit vaccines formulated in alhydrogel for human use has been impaired by the apparent inability to induce long-lasting immunological memory that provides protection against infectious aerosol plague challenge. In prior studies, alum-based Alhydrogel alone as an adjuvant has proven insufficient to induce durable antibody responses to plague vaccination (1, 45). Given these shortcomings, the search for additional adjuvants or presentations that can induce a lasting antibody response is critical for the success of effective plague vaccine development. The present study provides mechanistic details of the enhanced T and B lymphocyte responses in the germinal center and their association with improved antibody recall responses resulting from the incorporation of Alhydrogel-bound cytokines or CpG ODNs in the vaccine formulation. A recently published study demonstrates the antibody titer threshold for protection against significant lethal aerosol *Y. pestis* challenge (5). The anti-FIV antibody titers enhanced by either CpG- or cytokine-formulations that are shown in our present study fall above this threshold for protection against aerosol challenge, although additional challenge studies are needed to directly evaluate the responses induced by the vaccine formulations tested in our present study.

In order for vaccination to be successful, a vaccine must induce the generation of antibodies which protect against the target infection or antigen. This process depends upon a robust response within the germinal center (GC) of the lymph node. Germinal centers within draining secondary lymphoid organs provide critical environments for the selection of activated antigen-specific B cells and their differentiation into long-lived memory B cells and plasma cells. Tfh cells within the GC provide critical signals to sustain the GC response, promote the positive selection and affinity maturation of GC B cells, and provide the key factors to drive long-lived memory B cell (MBC) and plasma cell (PC) differentiation (30). Thus, Tfh cells are required for effective and long-lived antibody responses. Most vaccines in current use have been developed on an empirical basis, instead of using rational design principles. Understanding the basis for the induction of long-term immunological memory provides a basis for the development of more effective vaccines, and the field of Tfh cell biology is an emerging core platform in that approach. Rational vaccine design efforts are now incorporating GC Tfh and GC B cell analyses in draining lymphoid organs and seeking to enhance Tfh cell responses to promote improved development of immunological memory and to modulate the selection and breadth of vaccine-induced antibody responses (46–48). Finding ways to enhance Tfh cell responses during vaccination may improve the quantity of long-lived antigen-specific producing plasma cells and memory B cells, resulting in protection against infection for longer periods of time.

Surprisingly, despite the number of plague vaccine studies in rodent models, the dynamics and magnitude of GC B cell and Tfh cell responses to plague subunit vaccination have not been previously investigated. In this study, we have performed longitudinal analyses of germinal center B cell and Tfh cell responses in draining lymph nodes following plague vaccination and boosting, in order to examine these responses and determine whether they can be enhanced by the addition of novel adjuvants and whether there is a correlation with improved antibody titers at memory timepoints (day 126) and following recall after antigen rechallenge. Our study reveals that, in contrast to the relatively weak responses induced by F1V or F1+V vaccines formulated with Alhydrogel alone, CpG 1826 or IL-2 + GM-CSF cytokine adjuvants formulated with Alhydrogel induce robust Tfh and GC B cell responses that lead to higher and more durable antibody production, as well as a significantly improved recall responses following re-exposure to antigen. These alterations in both magnitude and duration of the GC response provide an increased window of opportunity for the generation of MBCs and plasma cells. Indeed, following antigen re-exposure to the rF1V antigen at day 126, mice previously immunized with these adjuvant combinations of Alhydrogel-bound CpG 1826 or IL-2/GM-CSF cytokines generated significantly higher antibody recall responses compared to mice immunized with vaccines formulated with Alhydrogel alone. This finding suggests that Alhydrogel-bound CpG 1826 and IL-2+GM-CSF supplementary adjuvants promote the generation of more long-lived MBCs that bear potential to rapidly generate plasmablast recall responses to induce high antibody titers following re-exposure to the rF1V antigen. However, the IL-2/GM-CSF cytokine formulated adjuvants resulted in higher titers compared to CpG immunized groups at some timepoints. Interestingly, the germinal center kinetics between CpG and IL-2/GM-CSF cytokine groups were different: cytokine adjuvant groups exhibited an early (day 7 and 14) enhanced GC B cell response following the primary immunization, while the CpG adjuvant groups exhibited significantly increased GC B and plasmablast responses following the booster that was given at day 16 post-immunization. These results emphasize that distinct mechanisms are in play for the priming of the immune system, and may result in differing memory B cell versus plasma cell fate programing. Given the distinct mechanisms and kinetics of the germinal center response induced by CpG versus IL-2/GM-CSF cytokine adjuvants, determining whether combining them into a single formulation results in additive or synergistic improvement of the germinal center response is an important question for future research. Additional studies are in progress to investigate how these adjuvants modulate GCs and how such changes affect MBC and long-lived PC numbers.

In addition to the quantity of antibodies produced, antibody isotype is important to engage effector mechanisms that are protective against *Yersinia pestis* infection. Based on the idea that the IgG_2a_ isotype promotes efficient opsonization of bacteria compared to other antibody isotypes (49), the vaccine-induced IgG2a titer and the IgG2a:IgG1 ratio have been important parameters to measure in plague vaccine studies (29, 50). Importantly, CpG or IL-2+GM-CSF formulated vaccines induced higher titers of F1V-specific IgG_2a_ and higher IgG2a:IgG1 ratios compared to alhydrogel-alone adjuvanted vaccines. The use of various CpG oligonucleotides (ODN) for enhancement of vaccination responses has been well studied. A recent study demonstrated that mice vaccinated with rF1V formulated with CpG 2006 provided improved protection against aerosolized *Y. pestis* challenge (29). The results reported in our paper support the findings of Birakov et al, and provide analyses of the immune response by CD4+ Tfh cells and GC B cells in the draining lymph nodes following vaccination which includes Alhydrogel-bound CpG 1826 or cytokines.

Examination of the levels of rF1V or LcrV-specific antibody induced by the IL-2/GM-CSF cytokine or CpG 1826 formulated vaccines in our study suggest that protection against a significant aerosol challenge in a murine model is likely achievable using these adjuvant-enhanced vaccines, based upon recent studies that establish threshold antibody titers associated with protection upon challenge (5). Furthermore, our data indicate that we are able to achieve a sufficiently high anti-LcrV titer that is associated with protection against a virulent F1-negative strain of *Y. pestis*, such as the non-encapsulated C12 strain (29). In addition to measuring the antigen-specific titers, we have also investigated the use of the AIM assay in order to provide an early indication of antigen-specific CD4+ cell responses in the draining lymph nodes as a predictor or vaccination efficacy. Our results suggest that use of the AIM assay to quantitate the presence of antigen-specific Tfh cells early during the immune response and throughout the duration of the GC reaction can provide an early predictor of vaccination efficacy and long-lasting immunity. This approach for using this assay in preclinical vaccine studies is under further development at the present time.

Our study provides a way forward for improved rational plague vaccine design, where modulating the function and differentiation of Tfh cells and GC B cells can serve to improve the durability of vaccine-induced protective antibody responses against plague and other infectious diseases.

## Data availability statement

The original contributions presented in the study are included in the article/**Supplementary Material**. Further inquiries can be directed to the corresponding authors.

## Ethics statement

The animal study was reviewed and approved by Institutional Animal Care and Use Committee at the University of Utah.

## Author contributions

DG and JSH conceived and designed experiments, performed experiments and analyzed data, provided supervision and oversight, and wrote and edited the paper. NN conceived and designed experiments, performed experiments and analyzed data, and wrote the paper. JL, NH, and GG performed experiments and analyzed data. EW, FF, and JNH provided supervision and oversight and edited the paper. All authors contributed to the article and approved the submitted version.

## Funding

This work was supported by the National Institutes of Health (NIH) grant R01 AI137238 (to JSH) and University of Utah Research Agreement Number 10052444 (to DG).

## Acknowledgments

The authors wish to thank USAMRIID for providing the rF1V antigen used in these studies through a Cooperative Research and Development Agreement with the University of Utah. Opinions, interpretations, conclusions and recommendations are ours and are not necessarily endorsed by the U.S. Army or the Department of Defense.

## Conflict of interest

Author FF was acting Scientific Director and was employed by CyTuVax B.V.. CyTuVax B.V. is the holder of a patent on the use of cytokines in vaccine formulations.

The remaining authors declare that the research was conducted in the absence of any commercial or financial relationships that could be construed as a potential conflict of interest.

## Publisher’s note

All claims expressed in this article are solely those of the authors and do not necessarily represent those of their affiliated organizations, or those of the publisher, the editors and the reviewers. Any product that may be evaluated in this article, or claim that may be made by its manufacturer, is not guaranteed or endorsed by the publisher.
