# Supplementary material for: The magnitude of the germinal center B cell and T follicular helper cell response predicts long-lasting antibody titers to plague vaccination
Source: Front Immunol. 2022 Oct 28;13:1017385. doi: 10.3389/fimmu.2022.1017385 (PMC9650111; doi:10.3389/fimmu.2022.1017385)
Supplement: Supplementary file 1 [file DataSheet_1.docx]

SUPPLEMENTARY MATERIAL


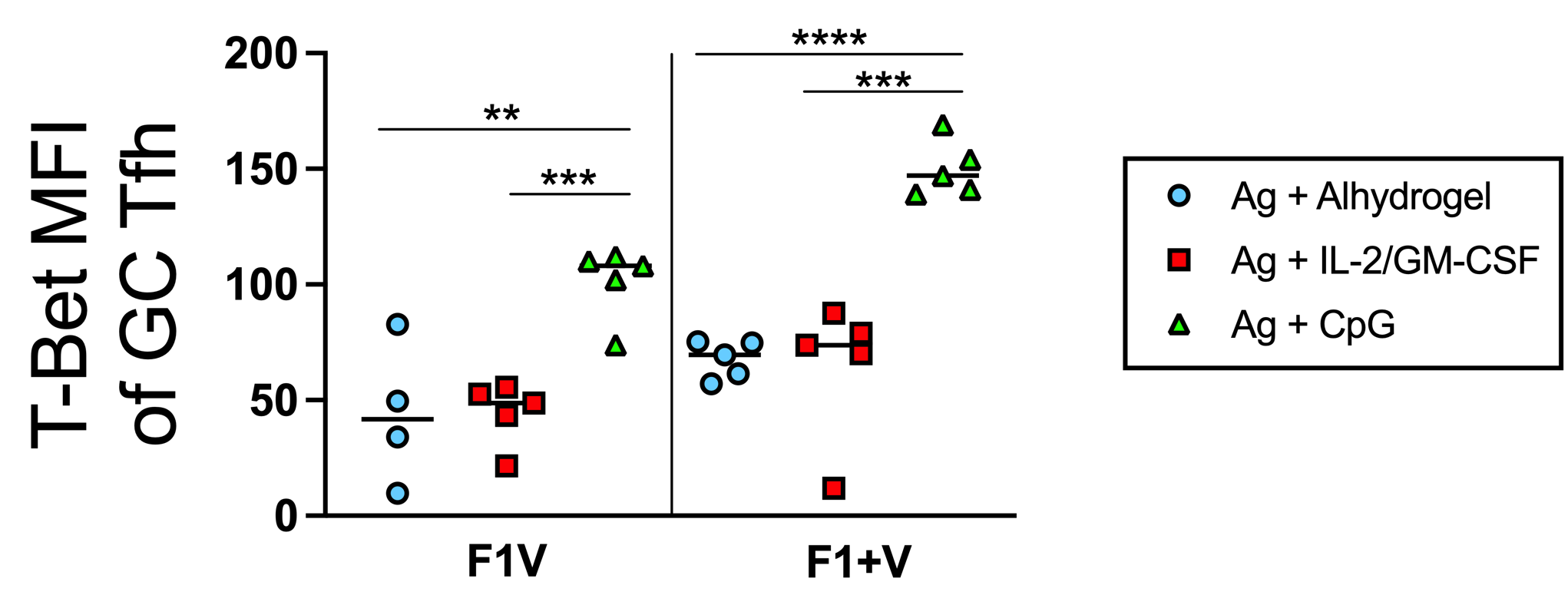


**Supplementary Figure 1: Tbet expression by germinal center Tfh cells 7 days post-primary immunization.** Analysis of Tbet expression by germinal center Tfh cells in the draining lymph nodes 7 days post-immunization. Charts show Tbet MFI gated on CXCR5+Bcl6+ GC Tfh cells. n=5 per group per group. Statistically significant p values were determined using a two-tailed unpaired Student t test; **p < 0.05, **p < 0.01, ***p < 0.001, ****p < 0.0001.*


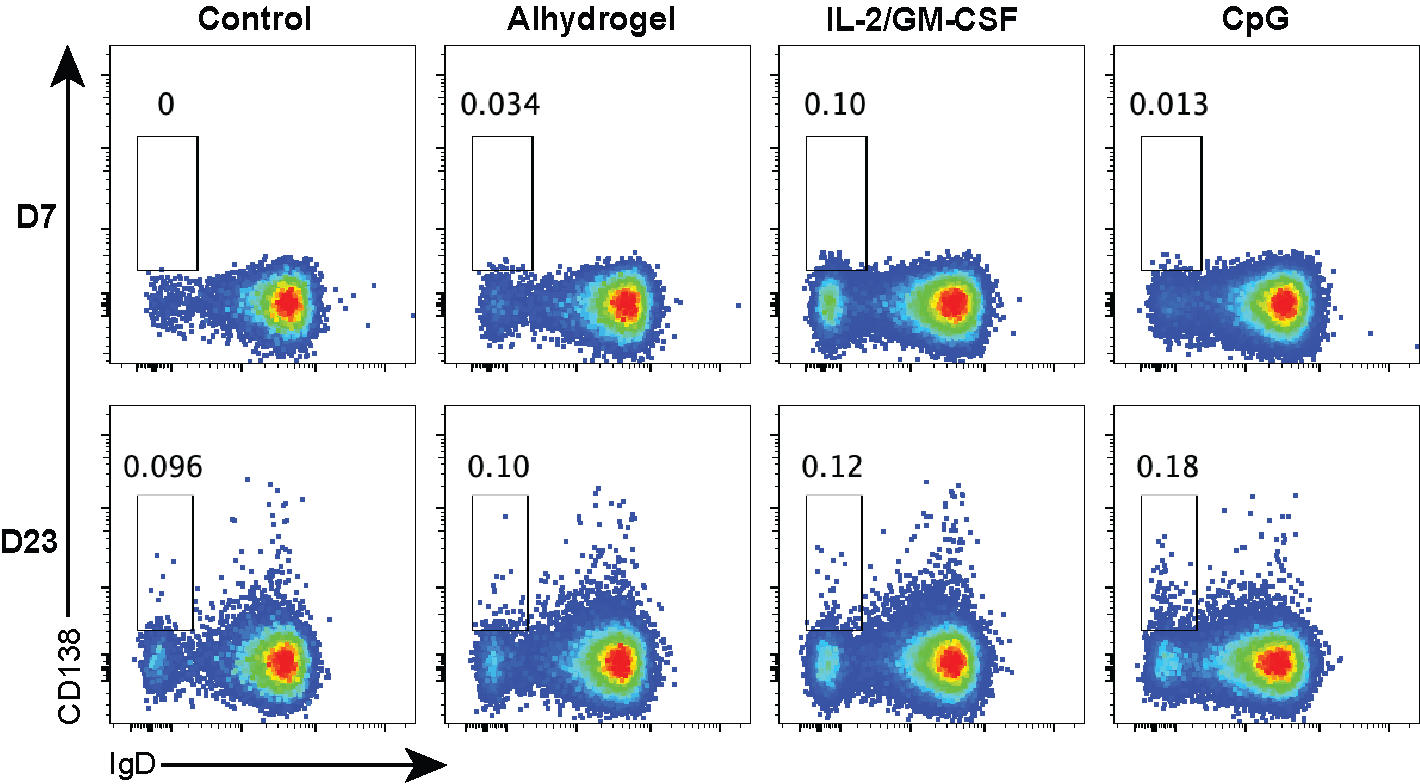


**Supplementary Figure 2: Gating Strategy for IgD^-^CD138+ plasmablasts.** Representative flow cytometry analysis CD19+ gated B cells with gate showing IgD-CD138+ plasmablasts in the draining lymph nodes at 7 and 23 days post-immunization. The numbers of plasmablasts are shown in Figure 5C.


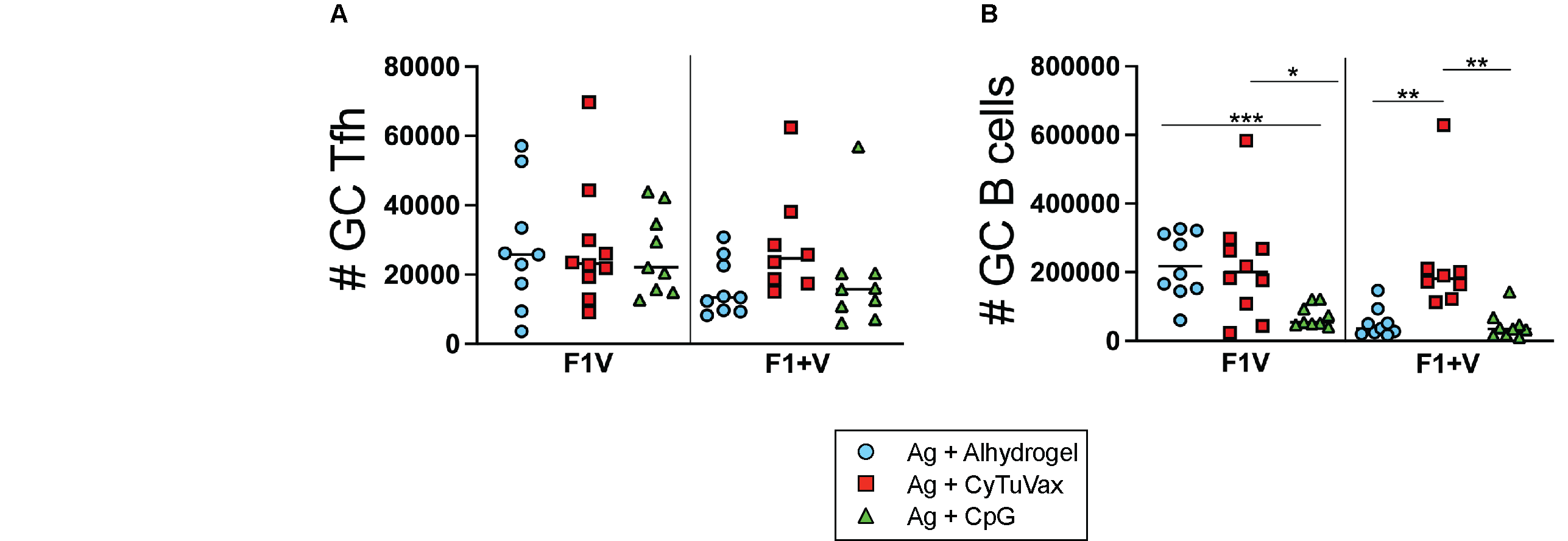


**Supplementary Figure 3: Germinal center Tfh and B cell responses in spleen 7 days post-re-exposure to F1V antigen.** Mice were immunized with F1V or F1+V antigen in combination with adjuvants on day 0 and given booster shots day 16. Mice were challenged by intraperitoneal injection with 10 μg of F1V antigen (no adjuvant) on day 126. One week later (day 133), splenocytes were analyzed by flow cytometry and quantified for (A) GC Tfh cell numbers; and (B) GC B cell numbers. Data shown are from one experiment and are representative of two independent experiments. Statistically significant p values were determined using a two-tailed unpaired Student T test; **p* < 0.05, ***p* < 0.01, ****p* < 0.001, *****p* < 0.0001.
